# Surface-Modified Extrinsic Semi-Distributed Interferometers for Fiber-Optic Refractive Index Detection and Biosensing

**DOI:** 10.3390/bios16050286

**Published:** 2026-05-15

**Authors:** Albina Abdossova, Toheeb Olalekan Oladejo, Sabira Seipetdenova, Marzhan Nurlankyzy, Aigerim Omirzakova, Aidana Bissen, Aliya Bekmurzayeva, Carlo Molardi, Cevat Erisken, Wilfried Blanc, Daniele Tosi

**Affiliations:** 1 School of Engineering and Digital Sciences, Nazarbayev University, Astana 010000, Kazakhstan; albina.abdossova@nu.edu.kz (A.A.); toheeb.oladejo@alumni.nu.edu.kz (T.O.O.); sabira.seipetdenova@nu.edu.kz (S.S.); marzhan.nurlankyzy@nu.edu.kz (M.N.); aigerim.omirzakova@nu.edu.kz (A.O.); aidana.bissen@nu.edu.kz (A.B.); carlo.molardi@nu.edu.kz (C.M.); cevat.erisken@nu.edu.kz (C.E.); 2 Laboratory of Biosensors and Bioinstruments, Center for Life Sciences, National Laboratory Astana, Nazarbayev University, Astana 010000, Kazakhstan; abekmurzayeva@nu.edu.kz; 3 INPHYNI, CNRS, Université Côte d’Azur, 06200 Nice, France; wilfried.blanc@univ-cotedazur.fr

**Keywords:** optical fiber sensors, interferometry, semi-distributed interferometer, refractive index sensor, thin-film coating

## Abstract

A semi-distributed interferometer is a low-reflectivity device with refractive index sensing capability, exploiting the random reflectivity of a nanoparticle-doped fiber to form a weak distributed cavity. In this work, we extend this concept to an extrinsic semi-distributed interferometer (ESDI), using an overlay made of polydimethylsiloxane (PDMS) around the fiber tip; this structure can then be surface-modified using a thin metallic film or a nanoparticle coating. We report gold-sputtered and gold-nanoparticle-coated ESDI structures for refractive index sensing capability, with the latter achieving superior performances with an average sensitivity of 62.8 dB/RIU (refractive index units) with resolution of 3.9 × 10^−5^ RIU over the range of 1.34790–1.35981. We also report a possible biological application using a biofunctionalized version of this probe for the detection of VEGF (vascular endothelial growth factor); the gold-sputtered probe achieves the highest sensitivity, 0.0565 dB for each 10× concentration increase, with 355 fM detection limit.

## 1. Introduction

Fiber optic sensors play a substantial role in modern digital monitoring technologies [1,2,3,4] as they enable single-point, multi-point, or distributed sensing along different geometries with compact probes [5,6,7], suitable for harsh and medical environments as well as for the tiniest packaging form factors [8,9].

Among these detection devices, refractive index (RI) sensors play a substantial role in medical and environmental applications. The measurement of RI in liquids [10,11] using a compact refractometer is a key asset in biosensing technologies [12]. As a stand-alone feature, it enables precise detection of the RI of unknown solutions with accuracy below 10^−3^ RIU (refractive index units) [13], and it can be linked to biomarkers encoded in the RI change; an example of this sensing capability is provided by the interrogation of glucose, suberic acid, or azelaic acid in a sweat mixture [14].

Refractometers are also the building block for label-free biosensors; by functionalizing the surface of the fiber with the use of bioreceptors such as antibodies or aptamers, the probe becomes sensitive to specific biomolecules that bind to their ligand over the fiber surface [15]. In this configuration, biosensors are a versatile technology: they have shown promising performance in terms of low detection limit and high sensitivity. Fiber optic biosensors have provided substantial diagnostic capability for cardiovascular [16], cancer [17,18], diabetic diseases [19], eye diseases [20], and fertility [21] biomarkers, considering the detection of proteins; latest works have extended this concept to larger molecules such as hormones [22,23], viruses [24], or even larger-than-wavelength targets such as recirculating cells [25].

In the analysis of fiber optic biosensors, the fiber modality and its geometry both play a substantial role [26]. Large-core, multimode fibers have been reported mostly for plasmonic biosensors, exploiting surface plasmon resonance, plasmonic resonances confined to localized nanoparticles, or lossy wave resonances [27,28]. These sensors have high sensitivity, encoded in plasmonic spectra measured at visible wavelengths and with spectral shift with sensitivity overcoming the 1000 nm/RIU figure, and for this reason, they are used as a benchtop device [29]. A successful example of this typology of sensor makes use of U-shaped bent large-core fibers, which have been integrated in sequential biosensing analysis [30,31]. However, in the context of in situ sensing, which requires the fiber probe to be inserted directly into the analyte [8] or in parenchymal tissue [32] to perform an online diagnostic, multimode fiber sensors have two substantial drawbacks. First, as light propagates in the outer fiber portion, sensors are very sensitive to fiber movements, which are very common in the in situ detection format, making the overall readout quite unstable. Secondly, plasmonic effects are inherently detectable only in the light transmitted through the sensor, which requires the detector to be placed after the fiber, looping the fiber back to the input; compact, in situ sensing probes would need instead a much more compact geometry of the fiber cabling.

A second approach would involve using a single-mode fiber as a platform to excite cladding modes. This methodology is the working principle of grating-based biosensors, which make use of the interaction between the light propagating in the cladding and the outer medium [26]. The two most popular approaches rely on tilted fiber Bragg gratings [33], which use a tilted Bragg grating to excite a comb of counter-propagating cladding modes [34], and long-period gratings that instead generate co-propagating modes in structures having several centimeters of length [35,36]. These systems make use of smaller fibers, typically single-mode telecom fibers, making probes more compact, but as the biosensing information is encoded in the multimode portion of the fiber cladding and in the transmitted light, they share the same practical issues of multimode fiber biosensors when it comes to in situ detection.

The use of single-mode fiber (SMF) propagation is therefore instrumental in the design of effective biosensors; however, a design based on SMF alone is not effective since the light remains confined within the fundamental mode and does not interact with the functionalized surface as we would require for a highly sensitive biosensor. A solution that has been consistently proposed is etching [37] or tapering [38] the sensing fiber, in order to force evanescent light to interact with the outer analyte. The etching approach has been proposed using Bragg gratings [37] or exposed core biosensors [39], while fiber tapers are used in harmonic gratings [16] or micro-resonators [40]. While these approaches succeed in solving the light propagation issues, providing compact reflective probes with mono-modality, the form factor of the sensors is typically very fragile, as the fibers are required to have a thin profile; therefore, when considering medical device packaging suitable for online diagnostic, it would be preferable to maintain the original form factor of the SMF fiber rather than compromising its mechanical properties.

Therefore, the use of single-mode fibers in the biosensing device is a key to enabling biosensors having both high performance and form factor suitable for in situ operation, provided that we maintain the mechanical strength of the fiber at the sensing tip. All-SMF biosensors have been reported using structures such as ball resonators [41], fiber-tip interferometers [42], and Fresnel probes [43], all of which operate in reflection mode with a compact design.

The semi-distributed interferometer (SDI) is an excellent candidate for biosensing, due to its inherent ease of fabrication, as well as high sensitivity and low detection limits [44]. In this work, we evolve the original SDI structure, adding an external layer of PDMS (Polydimethylsiloxane) around the original fiber [45]. This new extrinsic structure can then be surface-treated, using a thin gold (Au) film or gold nanoparticles (NPs).

**Figure 1:**
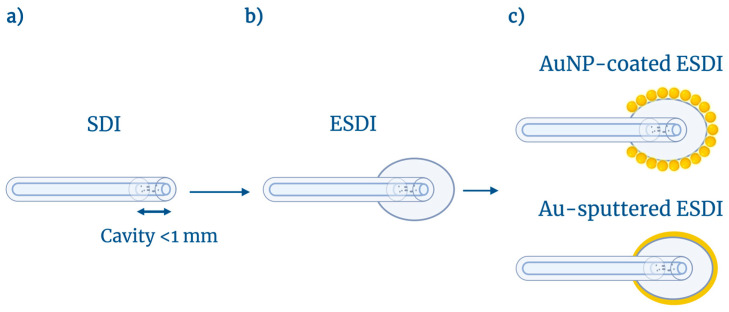
Schematic illustration of ESDI sensor fabrication: (**a**) bare SDI sensor; (**b**) ESDI sensor coated with a PDMS layer; and (**c**) AuNP-coated ESDI and Au-sputtered ESDI. Created with BioRender. Seipetdenova, S. (2026) https://BioRender.com/wij7c9h.

The concept of this work is displayed in Figure 1. The original SDI structure is formed by splicing a fiber with a high density of scattering centers to a SMF fiber, and then cleaving the tip to form a second mirror [42]; the two side mirrors form a cavity with very low finesse, interleaved by layers of scattering centers having a random reflection pattern. The second step, introduced in this work, involves coating the SDI sensor with a layer of PDMS. We label this device extrinsic SDI (ESDI), since the in-fiber format is complemented by the oval-shaped PDMS layer that protrudes out of the original fiber. The third step involves the treatment of the fiber surface, which can implement increased sensing features, forming a surface-modified ESDI. We report a thin-film Au-coating, which increases the sensitivity to biological analytes as reported in [46], as well as Au nanoparticles that augment the RI sensitivity. We validate the sensors in the context of refractive index detection, as well as in the detection of VEGF (vascular endothelial growth factor) cancer biomarker using a biofunctionalization of the probe [47], proving its effectiveness in biosensing of protein-based markers.

Table 1 below compares previous optical biosensors for VEGF detection in terms of sensing mechanism, recognition element, nanomaterials/platform, and detection limit. These systems employ different recognition elements, and their reported detection limits span a wide range.

## 2. Materials and Methods

### 2.1. Reagents and Biological Materials

Sulfuric acid, hydrogen peroxide, (3-Aminopropyl)trimethoxysilane (APTMS), methanol, polydimethylsiloxane (PDMS), AuNPs, sucrose, 11-Mercaptoundecanoic acid (MUA), ethanol, and phosphate-buffered saline (PBS) tablets were purchased from Sigma-Aldrich (Darmstadt, Germany); 1-ethyl-3-(3-dimethylaminopropyl)carbodiimide hydrochloride (EDC), N-hydroxysuccinimide (NHS), VEGF antibodies (PAA143Hu01) and VEGF proteins (RPA143Hu01) were purchased from Cloud-Clone Corp. (Wuhan, China), while methoxypolyethylene glycol-amine (mPEG-amine) was purchased from Thermo Scientific (Waltham, MA, USA).

### 2.2. Fabrication of the Fiber Optic Sensors

The ESDI sensors were developed through a splice and cleave approach that requires simple manual handling with telecom-grade fusion splicing equipment. The fabrication of the ESDI sensor began with the removal of the cladding using a fiber peeling plier, thereby exposing the uncoated fiber sections. The exposed fiber is then precisely cut with a Fujikura CT08 fiber (Fujikura, Koto City, Japan) cutter to ensure a straight, clean end. A pigtail (SMF) is also peeled and cut, ready to be spliced to the SDI. The two fiber segments (SDI and SMF) were spliced together using a standard telecom splicer (Fujikura 12-S) so that the cores of both fibers were perfectly matched. After that, a cavity was formed manually by cleaving the fiber at a very short distance of less than 1 mm using a fiber cleaver (Fujikura CT-08), forming a tip mirror [52]. All sensors used in the experiments were then calibrated for refractive index analysis, using 6 sucrose mixtures starting with 6 mL of 10% sucrose solution and by adding 400 μL of 40% sucrose solution subsequently and thereby covering an RI span of 0.01084 RIU, from 1.34761 to 1.35845.

### 2.3. Biofunctionalization

Functionalization is an integral part of the biosensor’s fabrication process. During this procedure, the sensor surface is modified, and a biorecognition element is attached to it, which makes it able to specifically bind to the target analyte. The optical fiber sensor functionalization process began with cleaning the surface of the fiber tip with a Piranha solution. The Piranha solution was prepared from a mixture of sulfuric acid (H_2_SO_4_) and hydrogen peroxide (H_2_O_2_) in a ratio of 4:1. The sensor was incubated in this solution for 15 min. In addition to cleaning the surface and removing impurities, the solution helps to increase the number of surface hydroxyl groups (-OH) [1]. The sensors were rinsed with distilled water (DI) and subsequently dried with nitrogen gas (N_2_) after Piranha cleaning. The dried fiber tip was immersed in a 1% solution of 3-aminopropyltrimethoxysilane (APTMS) in methanol for 20 min to functionalize the surface by attaching amino groups. These functional groups serve as a matrix for other functionalization layers. After silanization, the tips were rinsed with methanol and baked at 110 °C for 1 h to promote cross-linking between silane molecules, thereby stabilizing the silane monolayer and strengthening its attachment to the fiber surface [20,53]. The sensor tips were rinsed with DI water and left to dry at room temperature while the PDMS solution was prepared.

#### 2.3.1. PDMS Coating

The PDMS solution was prepared by mixing the base and curing agent at a 10:1 ratio. The mixture was stirred for 30 min to obtain a homogeneous and viscous solution. The sensors were reattached upside down, placed in a beaker, and secured with tape. PDMS was applied directly on the sensors’ tips using a small loop to create a small lens. The sensors were dried for 1 h at room temperature and then placed in the oven at 110 °C for another 1 h. After baking, the sensors were rinsed with DI water, allowed to dry at room temperature, and then calibrated. After calibration, the sensors were subjected to UV/ozone cleaning using the ProCleaner™ (Model 220) (Bioforce Nanosciences, Virginia Beach, VA, USA) for 20 min to effectively remove organic contaminants and increase the fiber tip’s surface energy by generating reactive oxygen species that break down surface residues and further form hydroxyl groups on the fiber surface [54,55]. Sensors with activated PDMS surface were again immersed in APTMS solution for 20 min and heated in the oven at 110 °C for 1 h. The sensors were rinsed with methanol after an hour to remove unbound silane molecules and impurities, ensuring a clean, uniform surface, and then dried with N_2_ gas to prevent contamination and moisture that could interfere with further processing. The sensors were baked in the oven for 1 h at 110 °C to promote cross-linking between silane molecules, stabilizing the silane layer and enhancing its adhesion and durability on the sensor surface, and also helped to remove residual solvents.

#### 2.3.2. Au-Sputtering and AuNP-Coating

One-half of the sensors were placed in an Au-sputtering machine (Q150T ES Plus, Quorum Technologies, Laughton, UK), which is a turbomolecular-pumped combined sputter coater and carbon evaporation system, for 10 min to coat the sensor tips with a 20 nm gold layer. This gold layer serves as a mirror, enhancing sensor sensitivity and providing a surface for further functionalization (e.g., binding biomolecules via thiol-gold chemistry). After sputtering, the sensors were placed in the oven at 80 °C for 1 h to remove moisture and residual solvents without damaging the fragile gold film. Furthermore, this low-temperature treatment enhances adhesion and stability, ensuring a consistent, long-lasting coating for optimal sensor performance.

The other half of the sensors was incubated in the AuNPs solution for 24 h. The synthesis of AuNPs was conducted using the citrate reduction method as presented by Turkevic et al. [56]. The AuNPs were synthesized by adding 2.0 mL of 34 mM trisodium citrate solution into a boiling solution, containing 0.5 mL of 1% hydrogen tetrachloroaurate (III) trihydrate and 50 mL of deionized water. The color of the solvent changed from bright yellow to dark violet in a few minutes and turned to ruby red after 15 min of stirring. The obtained solution was cooled at room temperature for the next 20 min and then cleaned by deionized water using a centrifuge operating at 15,000 rpm in 2 mL tubes. After incubation, sensors were washed with DI water and annealed at a temperature of 100 °C for 1 h.

#### 2.3.3. Antibodies Immobilization

**Figure 2:**
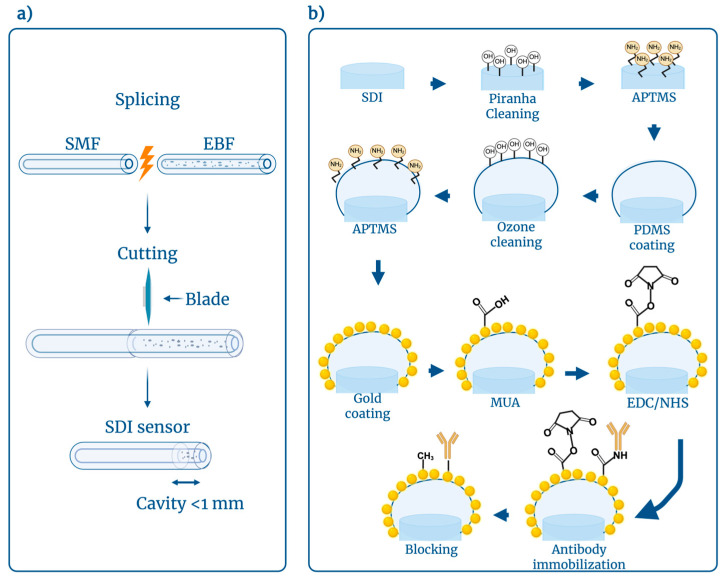
Schematic overview of the fabrication and complete functionalization of the ESDI sensor: (**a**) fabrication process of the SDI sensor; (**b**) functionalization of the ESDI sensor using AuNPs-coating and Au-sputtering, followed by immobilization in the biological recognition element for analyte detection. Created with BioRender. Seipetdenova, S. (2026) https://BioRender.com/pagtzgt.

After removal from the oven, both groups of sensors were washed with DI water and then calibrated following the same process as mentioned above. Washed sensors were submerged in 10 mL of a 9 mM solution of 11-mercaptoundecanoic acid (MUA) in absolute ethanol for 16 h at 4 °C to form a self-assembled monolayer (SAM) and expose the carboxyl groups to the sensor surface. The carboxyl groups (-COOH) at the other end of MUA are directed outward, but the thiol groups (-SH) make strong covalent connections with the gold surface. This produces a persistent, well-organized layer on the sensor surface that displays carboxyl functional groups, which are later required for the immobilization of biomolecules [57]. The sensor tips were rinsed with ethanol, and then with phosphate-buffered saline (PBS). This procedure was followed by bioconjugation activation of the surface by placing the sensor tips in a 1 mL solution containing 250 μL of 250 mM 1-ethyl-3-(3-dimethylaminopropyl) carbodiimide hydrochloride (EDC), 100 μL of 100 mM N-hydroxysuccinimide (NHS), and 650 μL of PBS. Incubation was carried out for 30 min. By forming an amine-reactive NHS ester, NHS stabilizes the unstable intermediate formed when EDC reacts with carboxyl groups [57]. This enables the sensor to operate as a particular biosensor for target detection by preparing the surface to covalently trap biomolecules (such as proteins, DNA, or antibodies) through stable amide bonds. Then, the sensor tips were rinsed with PBS and incubated with anti-VEGF antibodies (PAA143Hu01, Cloud-clone Corporation, San Diego, CA, USA) at 8 μg/mL for 1 h to immobilize the antibodies. After which, the sensor tips were rinsed with PBS to remove unimmobilized antibodies and then incubated in a 10% solution of methoxypolyethylene glycol-amine (mPEG-amine) for 30 min to block unreacted sites. Following blocking, the sensor tips were rinsed with PBS and then stored in a tube containing PBS to keep them stable and hydrated and stored at 4 °C until biomarker detection. The whole functionalization process is shown in Figure 2.

### 2.4. Experimental Setup

**Figure 3:**
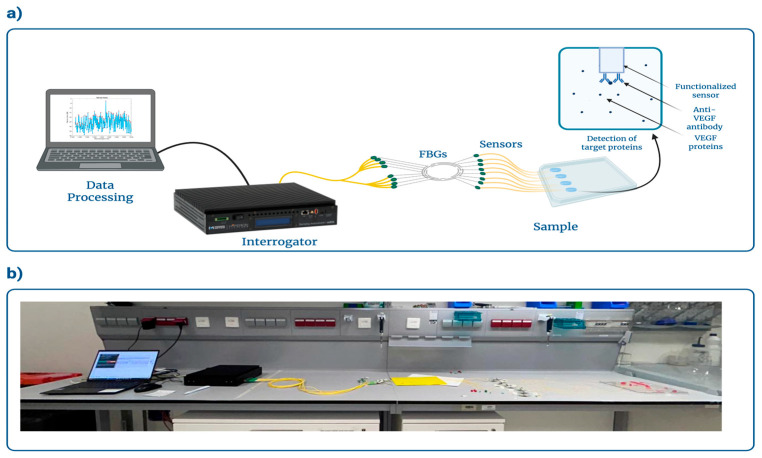
Overview of the experimental setup used for VEGF protein detection by ESDI biosensor consists of: (**a**) the schematic representation of the ESDI sensor and VEGF-antibodies interacting with the target of interest; (**b**) the experimental setup. Created with BioRender. Seipetdenova, S. (2026) https://BioRender.com/ggnciyu.

The sensors were used to detect vascular endothelial growth factor (VEGF) protein, which is an essential signaling protein that promotes angiogenesis, and a prominent biomarker linked with several diseases, including cancer, diabetic retinopathy and cardiovascular diseases [58,59]. First, a 1 μM solution of VEGF was prepared by mixing 4.5 μL of stock VEGF solution (with a concentration of 500 mg/mL) and 395.5 μL of PBS. Then, 5 concentrations of the protein were further made: 100 nM, 1 nM, 10 pM, 100 fM, and 1 fM; a PBS-only solution was used as a control. To make the various concentrations, the solution was diluted serially. A 100 nM solution was made by adding 40 μL to 360 μL of PBS. The solution was carefully mixed by resuspending, and 4 μL of the solution was taken and added to 396 μL of PBS to make a 1 nM solution. The same process was repeated to obtain the lower concentrations (10 pM, 100 fM, and 1 fM). After the protein preparation, the detection process commenced with the sensor tips placed in the protein solutions (see Figure 3). The protein solutions were detected from the smallest concentration to the largest concentration, starting with a PBS solution as a control. For a 1-sample spectral response, the data were recorded using a Micron Optics interrogator (Micron Optics si255, Luna Inc., Roanoke, VA, USA) (see Figure 3). A high-performance multi-channel sensing platform, the Micron Optics si255 optical interrogator is intended for accurate interrogation of fiber optic sensors in wavelength ranges up to 1460–1620 nm. With wavelength precision as high as 1 pm, the system can support scan rates ranging from 10 Hz to 5000 Hz, depending on configuration. It is ideal for sophisticated biochemical, biomedical, and engineering sensing applications needing high sensitivity and real-time performance since it is compatible with FBGs, LPGs, Fabry–Perot, and Mach–Zehnder interferometric sensors. The data was saved 30 times with an interval of 45 s. For all the surface-treated ESDI probes, we added a Fiber Bragg Grating, FBG (Technica S.A., Beijing, China), with Bragg wavelength 1550 nm and reflectivity ~90%. The FBG serves two purposes: it acts as a steep reflector, hence enabling the interrogation with a dynamic FBG analyzer, and it enables temperature compensation in case of thermal deviation from room temperature [14]. After all detections, the data was analyzed using the MATLAB software (R2025b).

### 2.5. Data Analysis

The spectra of the fiber optic sensors were processed using a feature extraction process, followed by a statistical analysis [44], using Matlab R2025B (Mathworks, Natick, MA, USA). Denoising was performed using a digital filter (Butterworth filter, 5th order, cut-off 0.01), which provides a good trade-off between spectral quality and noise reduction. From all interferometers’ spectra, the first step consisted of the removal of the Fiber Bragg Grating (FBG) spectral slice, and then identification of spectral features (peaks and valleys); this step was performed using a local maxima/minima identification, with prominence of 1 dB (SDI, ESDI, and AuNP-coated ESDI) or 0.02 dB (Au-sputtered ESDI).

To improve the accuracy of detection using all spectral features, for each measurement of RI or VEGF, we estimated the amplitude change (ΔA) for each identified spectral peak. Then, we used a Gaussian fit to match the probability density function (PDF) for each spectral intensity change; this way, we can track the average of the estimated PDF as the key marker for the intensity change.

For the refractometric measurements, we use three performance indicators: (1) reflectivity, measuring the average spectral levels of all identified peaks or valleys in reference conditions; (2) RI sensitivity, obtained by linear fit between the amplitude change (ΔA) and the refractive index change (Δn), estimated over a narrow RI range (0.0119 RIU), considering the mean values of the estimated Gaussian PDFs; and (3) fringe visibility (*FV*), evaluated as:

(1) $$FV=\frac{R_{P,av}-R_{V,av}}{R_{P,av}+R_{V,av}}$$

where *R_P_*_,*av*_ and *R_V_*_,*av*_ are the average reflectivity levels of peaks and valleys, respectively, in linear units. The choice of this RI range and incremental steps mimics the detection in biological samples and also allows a small-signal analysis using a linear approximation [60].

For biological measurement of VEGF, we evaluate three performance indicators reporting the capacity for protein detection: (1) sensitivity, evaluated performing a fit y = f(x), where x is the concentration of VEGF (molar units), *y* is the response (ΔA, in dB units), and f(x) is a log-linear fit similar to a previous SDI detection pattern [44]; (2) limit of detection (*LoD*), evaluated as in [61,62]:

(2) $$y_{LoD}=f^{-1}(y_{blank}+3\sigma_{max})$$

where *y_blank_* is the reference response for the blank sample, and *σ_max_* is the maximum, across all concentrations, of the standard deviation recorded over 10 consecutive samples after stabilization; and (3) response time, evaluated as the time for recording the 10–90% transition of the sensor response after changing VEGF concentration.

## 3. Results

### 3.1. Refractive Index Detection Using Au-Sputtered ESDI Sensors

**Figure 4:**
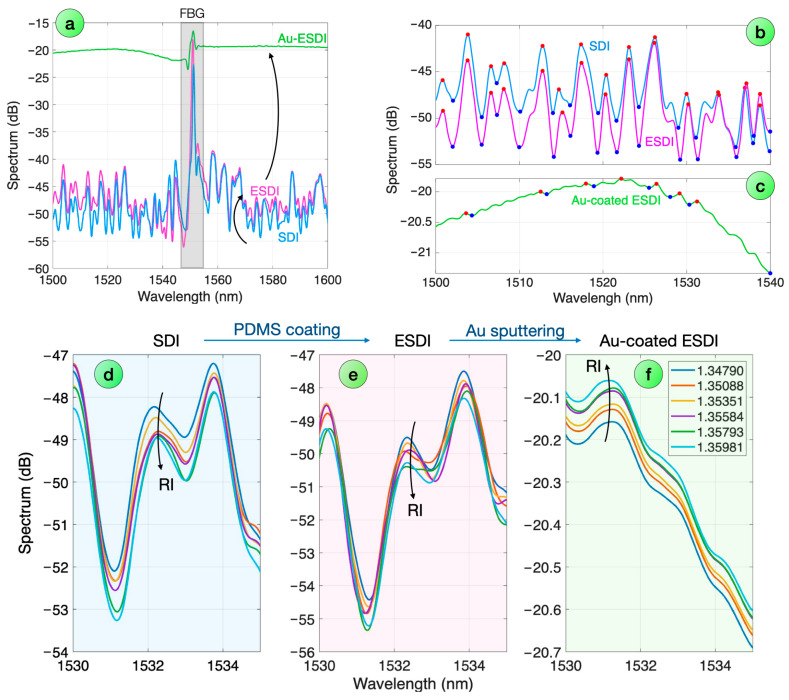
Spectra of an Au-sputtered ESDI probe throughout the fabrication process. (**a**) Spectrum of the probe across each fabrication step. (**b**,**c**) Spectral feature identification for the SDI and ESDI (**b**), and for the reflectivity-enhanced Au-sputtered probe (**c**), visualized over a 40 nm window; detected peaks and valleys are shown as red/blue dots. (**d**–**f**) Spectra obtained for each refractive index value, from 1.34790 to 1.35981, visualized over a 5 nm span, for each fabrication step: (**d**) SDI, (**e**) ESDI, and (**f**) Au-sputtered ESDI.

Figure 4 displays the spectra of an Au-sputtered ESDI probe across each fabrication step, and its refractive index dependence. The first chart displays the spectra of the interferometers visualized over a 100 nm window. We can observe that the initial SDI probe is characterized by spectral swings with a quasi-periodical pattern, as previously observed in experimental [44] and theoretical [63] works; in this sample, the spectrum has a low reflectivity, below −40 dB level, while the spectral valleys have a level within −55 to −50 dB. The spectral slice around 1550 nm features the FBG, which appears as a much larger reflector, and is discarded from the peak extraction feature. After the PDMS coating, we obtain the ESDI probe with an external layer around the SDI. The resulting spectrum does show a similar spectral pattern, with peaks and valleys maintaining the same wavelength, with minor changes to the spectral envelope. With the Au-sputtering, the reflectivity increases substantially, approaching −20 dB, as visualized by the steep rise in the spectral floor. We also observe that spectral peaks appear to smooth substantially, with a peak/valley difference inferior to 1 dB.

Figure 4b shows the spectral feature extraction (visualized over a 40 nm window) for the SDI/ESDI steps. The PDMS coating process maintains the pattern of peaks and valleys, with 14 instances of each feature observed in this spectral frame, having the same wavelength occurrence. The Au-sputtered ESDI, shown in Figure 4c, instead displays a different spectrum: as the reflectivity enhances and the fringe visibility lowers, spectral features can be detected only by lowering the peak prominence (to 0.02 dB). The envelope is much smoother, and a lower number of peaks and valleys can be detected (7 each). The wavelength pattern maintains a similar recurrence as the original SDI spectrum, with the misalignment mostly due to the spectral filtering, but we can see that the surface treatment is responsible for the spectral modification.

We show in Figure 4d–f the refractive index sensitivity for each of the spectra, displayed over a narrow 5 nm window for better visualization. For the SDI spectrum, we see that each spectral feature (peak/valley) appears to shift downwards in intensity for each incremental RI increase, following a Fresnel reflection pattern [64]. The sensitivity appears to increase in the spectral valleys, as observed in previous works and compatible with SDI modeling [63]. With the PDMS coating, the ESDI probe maintains a similar pattern, but the sensitivity appears to diminish, as witnessed by a less clear RI-sensitive pattern observed in correspondence with the spectral peaks. With the Au-sputtering, the Au-sputtered ESDI probe appears to have a clear RI sensitivity, but with the opposite sign as the spectra increase in reflectivity for each increment in surrounding RI.

**Figure 5:**
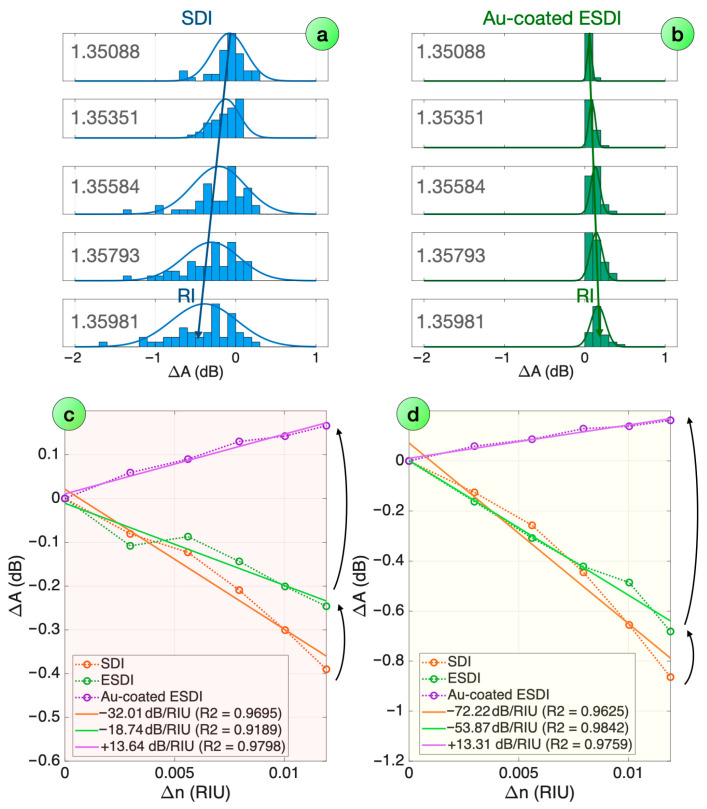
Statistical refractive index analysis for Au-coated ESDI probe. (**a**,**b**) Normalized PDF of spectral peak intensity change (histogram) and its Gaussian distribution fit (curve), reported for SDI (**a**) and Au-coated ESDI (**b**) probes. (**c**,**d**) RI sensitivity obtained by estimating the average of the Gaussian PDF fit for each RI value. Experimental data and the linear fitting are reported for (**c**) spectral peaks and (**d**) spectral valleys.

Since the RI sensitivity is encoded in each spectral feature, the statistical analysis is a key tool to improve the robustness of the detection method: rather than tracking the most significant feature, we can incorporate all the peaks/valleys detected in the process of feature extraction. This process is shown in Figure 5a,b: for each RI value, the histogram displays the PDF distribution of the spectral amplitude change (ΔA). We then perform a Gaussian distribution fit, obtaining an average value that follows the RI change. As shown in the previous figure, in the case of an SDI probe, we observe a progressive change towards a negative response, as the intensity decreases when the RI increases; the opposite trend is observed in the case of the Au-sputtered ESDI probe, where we observe a positive shift.

Using the statistical analysis, the RI sensitivity can then be estimated as in Figure 5c,d for each step of the probe fabrication, both in the case of spectral peaks and spectral valleys analysis. For the SDI probe, we can estimate the sensitivity as 32.01 and 72.22 dB/RIU, with a negative slope and a high linearity coefficient (>0.96). By forming an ESDI through the PDMS layer, we observe a decrease in sensitivity (18.74 and 52.87 dB/RIU, respectively), and the linearity coefficient for the peak detection decreases to 0.92. The final Au-coating process for the ESDI causes a change in the sensitivity (13.64 and 13.31 dB/RIU, respectively) and reverses the trend towards a positive slope.

This method for sensitivity estimation does not return the maximum possible sensitivity value, as this would be achieved by estimating the most significant spectral feature [60]; however, it results in a more robust detection in practical applications, as it would increase the tolerance to the noise and quality of spectral readings [44].

### 3.2. Refractive Index Detection Using AuNP-Coated ESDI Sensors

**Figure 6:**
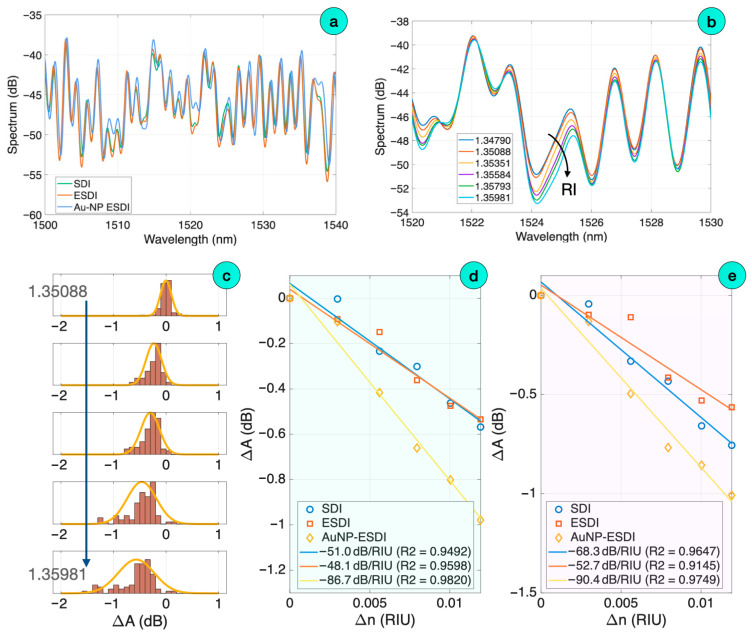
Spectra and refractive index sensitivity for an AuNP-coated ESDI probe. (**a**) Spectrum of the probe through the fabrication process (SDI, ESDI, and nanoparticle-coating), visualized over a 40 nm window. (**b**) Spectra of the AuNP-coated probe, for different RI values, are displayed over a narrow 10 nm window. (**c**) Normalized PDF of spectral peaks observed for each RI value, for the AuNP-coated probe, and its Gaussian PDF fitting. (**d**,**e**) Refractive index sensitivity, estimated for RI peaks (**d**) and valleys (**e**).

A similar analysis was performed for an AuNP-coated ESDI probe, as reported in Figure 6. The first chart displays the spectra of the probe through each fabrication step. The SDI probe has the typical random pattern observed in this type of chaotic interferometry [44], while the ESDI probe maintains a similar spectrum with peaks and valleys almost overlapping the original SDI envelope. The nanoparticle coating process, however, does not significantly impact the reflectivity: the observed increment in reflectivity is almost unnoticeable, and the spectrum maintains a similar envelope with no substantial changes. This is due to the fact that the distribution of nanoparticles along the PDMS coating does not substantially impact the overall sensitivity, and the spectral pattern remains unchanged through this process.

The refractive index dependence of optical spectra shows a similar pattern, as in Figure 6b,c. The spectra appear to decrease in intensity for the AuNP-coated probe, and this can be observed for each spectral feature. The statistical analysis shows that the PDF of spectral peaks shifts towards negative amplitude values when the RI increases.

Consistently, the RI sensitivity has a negative slope for each step of fabrication of the probe. The initial SDI sensitivity is 51.0 dB/RIU and 68.3 dB/RIU recorded for spectral peaks and valleys, respectively. When the PDMS coating is applied, forming an ESDI probe, the sensitivity decreases to 48.1 dB/RIU and 52.7 dB/RIU, respectively. When coating the ESDI with an AuNP layer, the sensitivity increases to 86.7 dB/RIU and 90.4 dB/RIU for peaks and valleys, respectively, improving also on the linearity coefficient (>97%).

### 3.3. Sensor Repeatability and Comparison

**Figure 7:**
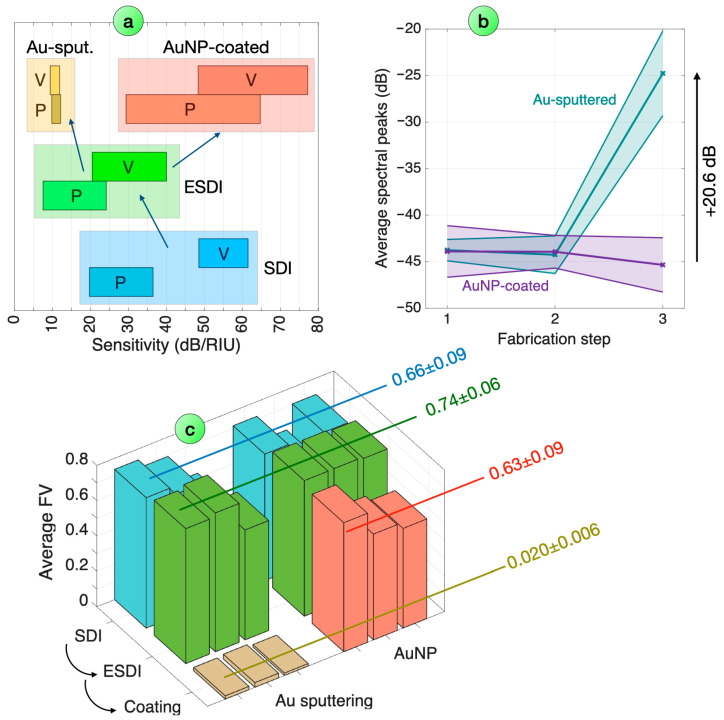
Comparison of the performance criteria for multiple surface-treated ESDI probes, for each fabrication step. (**a**) RI sensitivity (in absolute value), recorded for each fabrication step from bottom to top, recorded for peaks (P) and valleys (V). The boxes report the mean value ± standard deviation for all the sensing devices. (**b**) Average spectral peaks for Au-sputtered ESDI and AuNP-coated ESDI devices, for each fabrication step (1—SDI, 2—ESDI, and 3—surface-treated ESDI). Data are reported as average (markers) ± standard deviation. (**c**) Average fringe visibility, reported for each sensor sample across each fabrication step.

In order to evaluate the performance of multiple sensors, we performed the fabrication of six different ESDI probes, coating three of them with an Au layer and three with Au nanoparticles. The comparison of the sensors is graphically shown in Figure 7, while in Table 2, we report the full breakdown of reflectivity, FV, and sensitivity values at each fabrication step.

The reflectivity of the AuNP-coated probes appears to remain constant throughout the process, as we do not detect substantial changes throughout the fabrication process; conversely, the average increase in reflectivity recorded in Au-sputtered ESDI is 20.6 dB, with average spectral peaks reaching −19.7 dB in the most reflective sample.

The FV metric displays the width of the spectral pattern, and we also observe a different pattern between the coated devices. The average FV observed for SDI is 0.66 (0.09 standard deviation), and slightly increases to 0.74 (0.06 standard deviation) with the PDMS coating. We see a clear split in FV values through the surface coating: when using AuNP coating, the FV decreases marginally (0.63 mean value, 0.09 standard deviation), while the Au-sputtering process causes the standard deviation to drop over 30 times, to an average value of 0.020 (standard deviation 0.006).

The sensitivity values are also substantially dependent on the type of coating. SDI probes have an average sensitivity of 28.1 dB/RIU (peaks) and 55.0 dB/RIU (valleys). The application of the PDMS outer coating results in a decrease in sensitivity, with average values of 15.8 dB/RIU (peaks) and 30.2 dB (valleys). For AuNP-coated devices, the sensitivity increases to 47.0 dB/RIU (peaks) and 62.8 dB/RIU (valleys); for Au-sputtered devices, the sensitivity decreases to 11.0 dB/RIU (peaks) and 10.7 dB/RIU (valleys).

### 3.4. VEGF Detection with Biofunctionalized Probes

**Figure 8:**
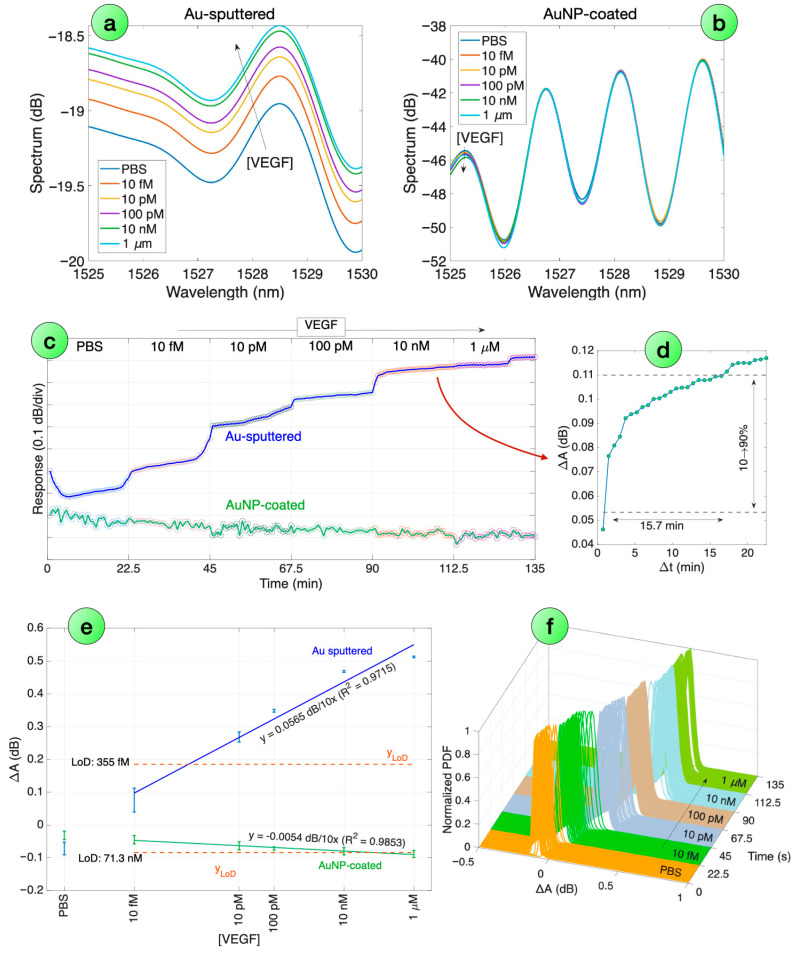
VEGF detection using biofunctionalized surface-modified ESDI probes. (**a**,**b**) Spectra of biofunctionalized Au-sputtered (**a**) and AuNP-coated (**b**) ESDI probes, for various VEGF concentrations (displayed over a 5 nm window). (**c**) Sensorgram displaying the intensity change timeline for the probes over the VEGF concentration increase. (**d**) Inset showing the response time of the biosensor. (**e**) Response of both biofunctionalized probes to VEGF concentration, using a log-linear fit and LoD estimation. Error bars show the standard deviation recorded over 10 values. (**f**) Normalized PDF reported for each measurement instance for the Au-sputtered ESDI biosensor.

VEGF detection was performed with an Au-sputtered ESDI and an AuNP-coated ESDI; both probes have been functionalized with VEGF-binding antibodies, with specificity validated in previous works [20]. The results of these biological measurements are reported in Figure 8.

We can observe that, as in line with other single-mode fiber biosensors [26], the biological sensitivity appears higher in the Au-sputtered ESDI sample, despite the lower RI sensitivity. As shown in Figure 8a,b, the spectra of this biosensor appear to have a quite clear dependence on VEGF, with the reflectivity increasing at each peak/valley in a perceivable way. The AuNP-coated biosensor instead follows the opposite trend, as the reflectivity decreases when the VEGF concentration increases. We applied the same analysis technique, performing the spectral feature extraction and PDF estimation with Gaussian fitting, for all measurement instances (45 s between each sampling); we report in Figure 8f the normalized Gaussian fit for the Au-sputtered biosensor, where we clearly see a shift towards the higher intensity values as the VEGF concentrations increase upwards.

The sensorgram reported in Figure 8c shows a real-time response for both probes. The Au-sputtered device shows a clear intensity change, up to about 0.6 dB from the reference in PBS. The pattern appears as a step-wise concentration, with a stabilization occurring within 15.7 min, as recorded at 10 nM concentration and displayed in Figure 8d. The trend observed for the AuNP-coated probe is lower in terms of intensity, about 0.1 dB intensity drop between the reference in PBS and the highest VEGF concentration, with a noisier pattern.

In Figure 8e, we report the detection chart, which displays the response measured for each concentration and the PBS reference, averaging over 10 consecutive measurements after reaching the response time; error bars show the excursion of the standard deviation. The probes have a log-linear pattern, with the response (in dB units) increasing linearly for each order of magnitude increment in the concentration, which enables a wide-range detection of the analyte. In this regard, the behavior is similar to other fiber optic biosensors [20], as well as electrochemical sensors, such as devices based on the Nernst principle [65]. The sensitivity, estimated over the 10 nM to 1 μM range, is 0.0565 dB for the Au-sputtered probe, and −0.0054 dB for the AuNP-coated sensor, both evaluated for a 10× increment in the VEGF concentration and with a high linearity coefficient (>97%).

Due to the higher sensitivity and cleaner sensorgram, the Au-sputtered probe provides a much lower detection limit of 355 fM, while the AuNP-probe has a LoD of 71.3 nM, closer to the upper part of the measurement range.

## 4. Discussion

The proposed results for a surface-treated ESDI probe provide interesting results for refractive index sensing and biosensing, and experimental implications for these measurement devices. At first, the addition of a PDMS layer around the fiber, which converts an SDI sensor into an extrinsic device, does not alter the original spectrum in a substantial way. This process does not provide a tangible improvement over the sensing performances (RI sensitivity), but the PDMS overlay provides an external encapsulation of the SDI sensor that might have experimental implications. At first, enlarging the sensing surface can be suitable for the detection of larger particles such as viruses [66], particularly in the case of large viral particles as occurring in biosafety-related projects [24], or cells having a size larger than the wavelength [67]. A second important factor is that a larger and round-shaped sensing surface is more suitable for packaging in medical devices for in situ detection, such as in the identification of tumor areas [68] or intra-venous, percutaneous, or sub-cutaneous packaging [9,69,70]: while a straight SDI sensor has a frontal reflective surface that can be more sensitive to motions during the measurements, particularly in the case of blood flow where blood pressure can periodically deform the sensor surface and the sensor-packaging alignment [71], a curved and larger surface is more resilient to motions and misalignments that occur in practical applications. In this regard, the ESDI concept, while preserving the performance factors of the original SDI device, improves the sensing geometry and packaging suitability for practical biosensing applications, approaching sensing concepts such as ball resonators [24,71] or ball-shaped lenses [72,73].

The performances in RI sensing and biosensing appear to diverge based on the type of surface treatment applied to the ESDI. For all the probes reported in this work, the experimental results consistently show that the AuNP coating improves the sensitivity for RI sensing, while preserving the reflectivity and fringe visibility of the original SDI structure, while Au sputtering increases the biological sensitivity (consistently with biological detection outcomes observed in single-mode fiber biosensors coated with metallic layer [57,74,75], and increments in the reflectivity while reducing the direct RI sensitivity and the FV).

In terms of RI detection, the AuNP-coated ESDI probes have an average sensitivity of 62.8 dB/RIU, recorded for the interrogation of spectral valleys, with an average of 66 spectral features identified over the spectral window. Considering that the noise floor of the FBG analyzer is 0.02 dB, we can therefore expect that the theoretical resolution is equal to 3.9 × 10^−5^, assuming that the noise recorded for each spectral feature is statistically independent of the other features.

Conversely, while the average sensitivity to RI for Au-sputtered ESDI probes is about six times lower (10.7 dB/RIU), the low fringe visibility reduces the number of detectable features. However, the large increment in reflectivity, over 20 dB, makes the probes also well detectable with standard analyzers such as static interrogators or high-resolution infrared spectrometers. This approach also shows higher biological sensitivity and is therefore preferred in functionalized biosensors.

## 5. Conclusions

In conclusion, we reported the design of a surface-treated extrinsic fiber optic device based on a semi-distributed interferometer, and its application to the detection of refractive index and biological analytes (VEGF). The proposed ESDI structure is based on a single-mode fiber SDI, coated with an outer PDMS layer to form an encapsulated structure with light propagating externally to the initial interferometer. The ESDI structure can then be surface-treated to enhance its sensing capability. We propose a surface treatment based on Au-sputtering, which increases the reflectivity of 20.6 dB on average and results in an augmented sensitivity in the biological detection, and a coating based on AuNPs, which in turn does not significantly alter the spectrum, but improves the refractive index sensing (62.8 dB/RIU average sensitivity). The biological detection of VEGF proposes a case scenario for biosensing, using a biofunctionalized ESDI probe: the Au-sputtered probe shows a sensitivity of 0.0565 dB, while the AuNP-coated probe has a lower sensitivity of 0.0054 dB, for each 10× VEGF concentration; the former probe can achieve a sub-picomolar detection limit (355 fM).

Future work will implement the surface-treated ESDI device for in situ biological detection, exploiting medical device packaging to deliver the probe in intravenous or subcutaneous form factors, and demonstrate the online sensing capabilities.

## Figures and Tables

**Table 1:** Optical sensors used for VEGF detection.

Sensor Type	Recognition Element	Nanomaterial or Platform	Detection Limit	Refs
WGM microcavity sensor	Antibody	Optofluidic chip	17.8 fg/mL	[48]
Fluorescence optical biosensor	Aptamer	DNA assembly system	3.5 pg/mL	[49]
Plastic optical fiber sensors	DNA aptamer	Au film	3 nM	[50]
Biolayer interferometry-based sensor	Peptide-nucleic acid aptamer pair (PNAP)	AuNPs	6 pM	[51]
Fiber optic sensor	Antibody	Magnesium Silicate Nanoparticles	26.6 fg/mL	[20]
Fiber optic sensor	Antibody	Au thin film and Au NPs	355 fM	Current work

**Table 2:** Performance of Au-sputtered and AuNP-coated ESDI sensors for RI detection, reporting three different sensors for each type. Fabrication steps: 1—SDI; 2—ESDI; and 3—surface-coated ESDI. Peak and valley reflectivity are averaged over all detected spectral features. RI sensitivity values are reported in absolute numbers.

Probe	Fabrication Step	Avg Peak Ref. (dB)	Avg Valley Ref. (dB)	FV	Peak Sens. (dB/RIU)	Valley Sens. (dB/RIU)
Au-sputtered (#1)	1	−44.8	−49.9	0.5276	32.0	72.2
	2	−46.4	−52.7	0.6214	18.7	53.9
	3	−19.7	−19.9	0.0137	13.6	13.3
Au-sputtered (#2)	1	−42.6	−49.9	0.6902	8.1	42.7
	2	−42.3	−51.5	0.7852	4.2	31.2
	3	−28.6	−28.9	0.0266	9.9	10.3
Au-sputtered (#3)	1	−43.9	−52.1	0.7373	9.4	54.8
	2	−44.1	−52.8	0.7632	7.0	23.0
	3	−25.9	−26.1	0.0187	9.4	8.4
AuNP-coated (#1)	1	−43.1	−50.7	0.7066	38.6	41.0
	2	−45.3	−53.9	0.7590	11.0	12.0
	3	−47.3	−52.8	0.5634	35.5	65.1
AuNP-coated (#2)	1	−47.0	−52.6	0.5718	29.7	51.0
	2	−44.5	−53.3	0.7678	5.9	8.8
	3	−46.8	−52.8	0.5983	18.9	32.9
AuNP-coated (#3)	1	−41.6	−49.5	0.7165	51.0	68.4
	2	−42.0	−50.8	0.7672	48.1	52.7
	3	−42.0	−50.1	0.7298	86.7	90.4

## Data Availability

The data reported in this work are available from the authors upon reasonable request.
